# Bidirectional joint line convergence angle changes and their preoperative predictors 12 months after medial closing‐wedge distal femoral osteotomy for valgus knees: A single‐centre exploratory study

**DOI:** 10.1002/jeo2.70975

**Published:** 2026-11-03

**Authors:** Yuki Kato, Ken Ichikawa, Hiroki Matsui, Shuzo Takazawa, Shunsuke Koga, Shin Yamada, Asami Kiuchi, Akira Ryu, Hiroshi Ohuchi

**Affiliations:** ^1^ Joint Preservation and Reconstruction Center Kameda Medical Center Kamogawa Chiba Japan; ^2^ Department of Sports Medicine Kameda Medical Center Kamogawa Chiba Japan; ^3^ Clinical Research Support Office Kameda Medical Center Kamogawa Chiba Japan; ^4^ Department of Clinical Epidemiology and Health Economics, School of Public Health The University of Tokyo Tokyo Japan

**Keywords:** coronal alignment, joint line convergence angle, medial closing‐wedge distal femoral osteotomy, preoperative predictors, valgus knee

## Abstract

**Purpose:**

To characterize the signed 12‐month change in joint line convergence angle (JLCA) after medial closing‐wedge distal femoral osteotomy (MCWDFO) for valgus knees and assess associations with preoperative factors, particularly its preoperative value.

**Methods:**

This retrospective single‐centre cohort included 62 knees treated with MCWDFO between October 2017 and December 2024 without additional osteotomy or ligament repair or reconstruction. The primary outcome was signed change (12‐month postoperative minus preoperative JLCA) on weight‐bearing full‐length radiographs. Univariable and multivariable linear regression analyses were performed. The primary model included preoperative JLCA, mechanical lateral distal femoral angle (mLDFA), mechanical medial proximal tibial angle (mMPTA), age, sex and body mass index (BMI). Cluster‐robust standard errors accounted for within‐patient dependence (eight bilateral patients). Internal performance was assessed using patient‐grouped 10‐fold cross‐validation (50 repeats), pooling out‐of‐fold predictions within each repeat.

**Results:**

Preoperative JLCA was −1.13 ± 2.04° and the 12‐month value was −0.56 ± 1.61°, with postoperative values more concentrated near 0°. Mean signed change was 0.57 ± 1.90° (median, 0.3°; range, −4.6° to 6.9°). Preoperative JLCA showed the strongest association with signed change (coefficient, −0.56; 95% confidence interval, −0.78 to −0.34; *p* < 0.001). mMPTA was also independently associated, whereas mLDFA, age, sex and BMI were not. Primary and cross‐validated coefficients of determination were 0.602 and 0.474; cross‐validated root mean squared and mean absolute errors were 1.36° and 1.10°.

**Conclusions:**

Signed change showed substantial between‐knee variability despite a small mean change. The association with preoperative JLCA should be interpreted cautiously because of mathematical coupling. The model showed moderate internal predictive performance and remains exploratory rather than a precise individual‐level prediction tool. External validation and formal radiographic reliability assessment are required before clinical implementation.

**Level of Evidence:**

Level III, retrospective cohort study.

## Graphical abstract

In 62 valgus knees from 54 patients, signed joint line convergence angle changes 12 months after medial closing‐wedge distal femoral osteotomy showed substantial between‐knee variability despite a small mean change. The prediction model remains exploratory and should not be used to determine patient‐specific correction angles. The radiographs are illustrative preoperative and postoperative images. JLCA, joint line convergence angle; MCWDFO, medial closing‐wedge distal femoral osteotomy; mMPTA, mechanical medial proximal tibial angle; R², coefficient of determination; SD, standard deviation.

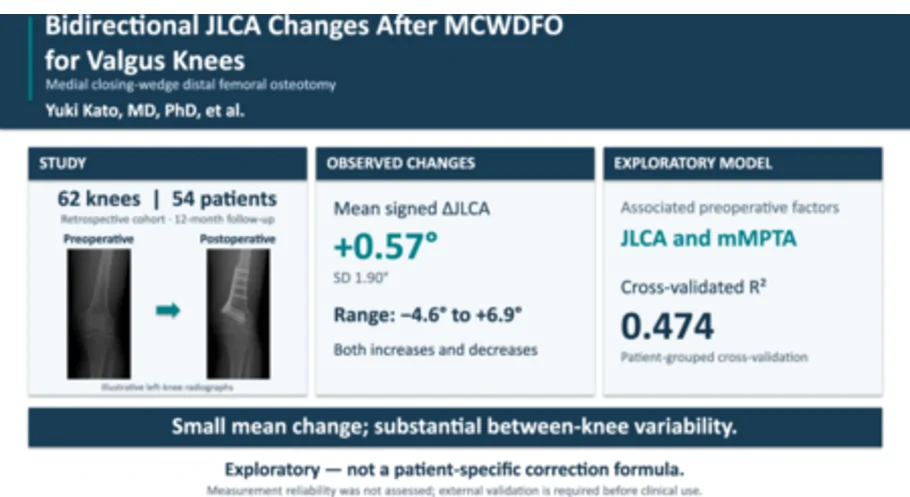

**Abbreviations**
ACI: autologous chondrocyte implantation
AIC: Akaike information criterion
*B*
: unstandardized regression coefficient
BMI: body mass index
BMS: bone marrow stimulation
CCI: Charlson Comorbidity Index
CI: confidence interval
CR2: cluster‐robust variance estimator with a small‐sample correction
DFO: distal femoral osteotomy
DLO: double‐level osteotomy
HKAA: hip–knee–ankle angle
HTO: high tibial osteotomy
JLCA: joint line convergence angle
JLO: joint line obliquity
KL: Kellgren–Lawrence classification
LCL: lateral collateral ligament
LCWDFO: lateral closing‐wedge distal femoral osteotomy
LOWDFO: lateral opening‐wedge distal femoral osteotomy
MAE: mean absolute error
MCWDFO: medial closing‐wedge distal femoral osteotomy
mLDFA: mechanical lateral distal femoral angle
mMPTA: mechanical medial proximal tibial angle
MOWHTO: medial opening‐wedge high tibial osteotomy
OATS: osteochondral autograft transplantation
REDCap: Research Electronic Data Capture
RMSE: root mean squared error
SD: standard deviation
signed ΔJLCA: 12‐month postoperative JLCA minus preoperative JLCA
SSE: sum of squared prediction errors
SST: sum of squared deviations of observed signed ΔJLCA from its overall mean
VIF: variance inflation factor
%MA: percentage of the mechanical axis

## INTRODUCTION

Medial closing‐wedge distal femoral osteotomy (MCWDFO) is an established joint‐preserving procedure for valgus knees with lateral compartment pathology, and recent reports have demonstrated favourable clinical and radiographic outcomes in pathologic valgus knees [14]. However, excessive correction should be avoided in closing‐wedge distal femoral osteotomy, and stable postoperative outcomes require preoperative planning that considers not only the magnitude of bony correction but also the intra‐articular deformity that ultimately determines postoperative weight‐bearing alignment [21].

The joint line convergence angle (JLCA) is an important radiographic parameter that reflects intra‐articular deformity, and most of the available evidence has been derived from varus knees. In varus knees, JLCA has been shown to correlate with intra‐articular deformity and the severity of medial compartment degeneration [12, 25]. Preoperative soft‐tissue laxity and larger correction angles have been reported to contribute to postoperative JLCA changes and overcorrection [9, 10], and a high preoperative JLCA has been identified as a predictor of postoperative alignment outliers [16, 24]. In addition, JLCA‐adjusted preoperative planning has been shown to improve correction accuracy in high tibial osteotomy (HTO) and double‐level osteotomy (DLO) [1, 6].

By contrast, the literature on JLCA in valgus knees is far more limited than that in varus knees. Although a preliminary report on DLO for valgus deformity is available [8], studies specifically focusing on intra‐articular alignment changes after distal femoral osteotomy (DFO) with JLCA as the primary topic remain scarce. Onorato et al. reported postoperative changes in JLCA and joint line obliquity (JLO), as well as an exploratory tipping point in valgus cases [17]; however, their cohort included both varus and valgus knees, and the surgical techniques were heterogeneous, including MCWDFO, lateral opening‐wedge DFO (LOWDFO) and lateral closing‐wedge DFO (LCWDFO). Furthermore, ΔJLCA was evaluated as an absolute difference, and the reported tipping point was positioned as an exploratory/descriptive threshold rather than a predictive cutoff. Therefore, both the distribution of signed ΔJLCA from preoperative assessment to 12 months after MCWDFO for valgus knees and the extent to which it can be estimated from preoperative factors—particularly preoperative JLCA—remain insufficiently understood.

It was hypothesized that signed ΔJLCA from preoperative assessment to 12 months postoperatively would vary substantially among knees and that its magnitude would be associated with preoperative JLCA. It was further hypothesized that other preoperative radiographic factors reflecting the location and severity of valgus deformity might also be associated with signed ΔJLCA. Accordingly, the primary objective was to characterize the distribution of signed ΔJLCA, and the secondary objective was to develop an exploratory model using preoperative radiographic and clinical factors, with particular emphasis on preoperative JLCA.

## METHODS

### Study design

This study was designed as a single‐centre retrospective observational cohort study of consecutive knees treated with MCWDFO for valgus deformity. Signed JLCA change from preoperative assessment to 12 months postoperatively and its associations with preoperative factors were evaluated.

### Setting

This study was conducted at a high‐volume tertiary referral centre specializing in joint‐preserving knee surgery in Japan. The institution performs approximately 150 around‐knee osteotomies annually. Clinical data, including patient demographics and radiographic parameters, are routinely collected in clinical practice and integrated into an institutional database managed using REDCap (Research Electronic Data Capture).

### Participants

Consecutive knees treated with MCWDFO for valgus knee deformity at the participating institution between October 2017 and December 2024 were retrospectively reviewed. Eligible knees underwent MCWDFO without an additional osteotomy or concomitant ligament repair or reconstruction. Concomitant cartilage, meniscal and lateral retinacular procedures were permitted. The inclusion criteria were (1) valgus knee deformity defined as a preoperative hip–knee–ankle angle (HKAA) ≥ 0° and (2) treatment with MCWDFO during the study period. The exclusion criteria were as follows: (1) concomitant HTO or DLO, (2) a history of intra‐articular fracture surgery, (3) a history of re‐osteotomy of the femur or tibia, (4) a history of lateral collateral ligament (LCL) repair or reconstruction and (5) inability to confirm, in the institutional database, the clinical data required for analysis, including preoperative and 12‐month postoperative weight‐bearing full‐length lower‐extremity radiographs necessary for evaluation of the JLCA and lower‐limb alignment parameters.

### Surgical technique

MCWDFO for valgus knees was performed through a medial approach to the distal femur. Using a subvastus approach, the vastus medialis was elevated anteriorly to expose the medial aspect of the distal femur. The correction angle was determined preoperatively on standing full‐length anteroposterior radiographs of the lower extremity, with a target HKAA of 0° to −1° (neutral to 1° of varus). No JLCA‐based adjustment was applied to the planned correction angle. The same target HKAA range was used for all knees, irrespective of cartilage status or concomitant procedures. A biplanar MCWDFO was performed according to the preoperative plan. The osteotomy site was closed, and correction was achieved using a DFO plate (Osferion BioMaterial Co., Ltd.) and a reduction screw. Coronal alignment was then assessed using an alignment rod with the patient in the supine position. Definitive fixation was completed after neutral to slight varus alignment had been confirmed.

### Variables

The primary outcome was signed ΔJLCA, calculated as the 12‐month postoperative JLCA minus the preoperative JLCA on weight‐bearing full‐length lower‐extremity radiographs; positive and negative values indicated increases and decreases in JLCA, respectively. JLCA was defined as positive when the medial joint space was narrowed and negative when the lateral joint space was narrowed.

The explanatory variables included preoperative JLCA, preoperative HKAA, preoperative mechanical lateral distal femoral angle (mLDFA), preoperative mechanical medial proximal tibial angle (mMPTA), preoperative JLO, age, sex and body mass index (BMI). JLO was calculated as mMPTA + mLDFA [13]. For the multivariable analysis, preoperative JLCA, preoperative mLDFA, preoperative mMPTA, age, sex and BMI were selected as candidate independent variables based on prior literature and clinical relevance.

Preoperative HKAA and JLO were excluded from the multivariable model because both are mathematically derived from mLDFA, mMPTA and/or JLCA, which would introduce collinearity if entered together with these component parameters.

Preoperative JLCA has been reported to be an important factor associated with postoperative JLCA change, correction error and alignment outliers in osteotomy for varus knees [1, 9, 10, 16]. Preoperative mLDFA and mMPTA were selected to separately capture the femoral‐ and tibial‐side contributions to coronal alignment, given their relevance to osseous deformity severity and to the association between femoral correction and intra‐articular alignment changes reported after DFO for valgus knees [17]. Age, sex and BMI were included in the model as clinically important background covariates for adjustment.

### Data sources and measurement

Clinical data, including patient demographics and radiographic parameters, were extracted from an institutional database managed using REDCap. Preoperative planning data and perioperative information were collected from the electronic medical records and preoperative planning records.

Radiographic assessments were performed using standing full‐length anteroposterior radiographs of the lower extremities obtained preoperatively and at the routine 12‐month postoperative follow‐up. Patients were instructed to distribute their weight equally between both lower limbs. Care was taken to avoid excessive internal or external rotation, using a forward‐facing patellar position as a positioning guide when anatomically feasible. All radiographs were obtained by radiologic technologists at the participating institution. A reference marker was included in the imaging field for size calibration in mediCAD (mediCAD Hectec GmbH; distributed in Japan by TOYO Corporation). The evaluated parameters included the HKAA, percentage of the mechanical axis (%MA), mLDFA, mMPTA, JLCA and JLO. Preoperative and postoperative radiographic measurements were performed as part of routine clinical practice by two physician assistants from the participating department who were experienced in lower‐limb alignment assessment using mediCAD. The same predefined measurement definitions and anatomical landmarks were applied at both time points. The assessors were not blinded to radiographic timing (preoperative vs. postoperative).

Previous studies have reported good interobserver reliability for JLCA measurements using mediCAD on long‐leg radiographs, supporting its use for radiographic alignment assessment [7, 20]. Formal intraobserver and interobserver reliability and absolute agreement were not assessed in this cohort.

### Bias

To minimize selection bias, all consecutive eligible knees treated with MCWDFO for valgus knees at the participating institution during the study period were included. To reduce information bias, patient demographics and clinical data were extracted from an institutional database that is routinely populated during clinical practice and managed using REDCap. Radiographic assessments were performed on standardized weight‐bearing full‐length lower‐extremity radiographs according to a predefined measurement protocol, as described above. However, the assessors performing the radiographic measurements were not blinded to the timing of the radiograph (preoperative vs. postoperative) in this study, which may have introduced measurement bias.

### Study size

This exploratory retrospective study was designed to describe signed ΔJLCA from preoperative assessment to 12‐month follow‐up after MCWDFO in the defined study cohort. Because previous reports on JLCA changes in a homogeneous MCWDFO cohort for valgus knees are limited, no formal sample size calculation was performed a priori. Therefore, all consecutive eligible knees treated at the participating institution between October 2017 and December 2024 were included in the analysis. The final sample size was determined by the number of eligible knees and the availability of the radiographic and clinical data.

### Quantitative variables

Continuous variables were analysed as continuous measures. Age, BMI, HKAA, %MA, mLDFA, mMPTA, JLCA and JLO were treated as quantitative variables. HKAA was defined as positive for valgus alignment and negative for varus alignment, whereas JLCA was defined as positive when the medial joint space was narrowed and negative when the lateral joint space was narrowed. JLO was calculated as the sum of mMPTA and mLDFA. The primary outcome, signed ΔJLCA, was calculated as the 12‐month postoperative JLCA minus the preoperative JLCA; positive values indicated an increase in postoperative JLCA, whereas negative values indicated a decrease. Changes in radiographic parameters were calculated as the 12‐month postoperative value minus the preoperative value. In the primary analyses, unnecessary dichotomization or categorization was avoided, and all continuous variables were analysed as continuous measures.

### Statistical methods

Statistical analyses were performed using R version 4.6.1 (R Core Team). Continuous and categorical variables were summarized as mean ± standard deviation (SD) and frequencies (percentages), respectively.

Associations between signed ΔJLCA and each candidate predictor were examined using univariable linear regression. The primary multivariable model included preoperative JLCA, mMPTA and mLDFA, together with age, sex and BMI. HKAA and JLO were excluded to avoid collinearity arising from their mathematical dependence on these radiographic parameters. Variance inflation factors (VIFs) < 5 were considered acceptable.

Because 8 of the 54 patients contributed bilateral knees (16 of 62 knees), cluster‐robust (CR2) standard errors with Satterthwaite degrees of freedom and clustering by patient were used to calculate 95% confidence intervals (CIs) and *p* values for all regression coefficients. Point estimates, *R* ^2^, adjusted *R* ^2^ and the Akaike information criterion (AIC) were obtained from the corresponding ordinary least squares models.

A sensitivity analysis used the same predictors with 12‐month postoperative JLCA as the outcome. Bidirectional AIC‐based stepwise selection was applied to the full model as a supplementary analysis; the prespecified full model remained primary. Additional sensitivity analyses examined each concomitant procedure separately using linear regression with signed ΔJLCA as the outcome.

Internal predictive performance was assessed using patient‐grouped 10‐fold cross‐validation (50 repeats, fixed random seed), keeping both knees from each patient in the same fold. Within each repeat, out‐of‐fold predictions for all 62 knees were pooled before calculating *R* ^2^, root mean squared error (RMSE) and mean absolute error (MAE); these metrics were then averaged across repeats. *R* ^2^ was calculated as 1 − SSE/SST, where SSE was the sum of squared prediction errors, and SST was the sum of squared deviations of observed signed ΔJLCA from its overall mean. The full and JLCA‐only models were evaluated using identical cross‐validation splits, and within‐repeat differences in pooled *R* ^2^, RMSE and MAE were summarized across repeats to assess incremental predictive value. A two‐sided *p* value < 0.05 was considered significant for regression analyses.

## RESULTS

### Participants

Between October 2017 and December 2024, 68 knees treated with MCWDFO for valgus deformity were screened for eligibility (Figure 1). One knee was excluded for concomitant DLO (*n* = 1), leaving 67 knees. Five further knees were excluded for previous surgery: open reduction and internal fixation for an intra‐articular fracture (*n* = 4) or knee osteotomy (*n* = 1). None were excluded for prior LCL repair or reconstruction (*n* = 0). The final analysis included 62 knees from 54 patients, including 8 with bilateral knees (16 knees). All had complete preoperative and 12‐month postoperative radiographic and clinical data; no knees were excluded for missing study variables.

**Figure 1:**
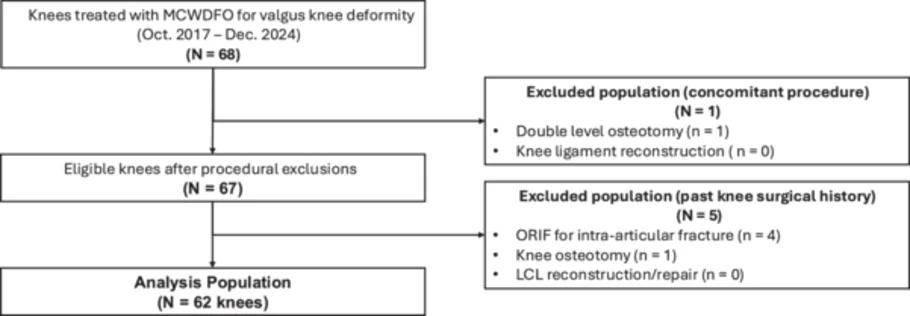
Patient selection flowchart. All 62 knees in the analysis population had complete preoperative and 12‐month postoperative radiographic and clinical data. LCL, lateral collateral ligament; MCWDFO, medial closing‐wedge distal femoral osteotomy.

### Cohort characteristics

Cohort characteristics are summarized in Table 1. The mean age was 54.2 ± 11.5 years, and the mean BMI was 23.7 ± 3.0 kg/m^2^. Twenty knees (32.3%) were from male patients, and 25 (40.3%) were left‐sided. Preoperative Kellgren–Lawrence (KL) Grade 2 was the most common (27 knees, 43.5%), followed by Grade 3 (24 knees, 38.7%). The mean operative time was 184.4 ± 54.4 min, and the mean intraoperative blood loss was 166.8 ± 166.7 mL. Concomitant procedures included lateral retinacular release (42 knees, 67.7%), osteochondral autograft transplantation (OATS; 50 knees, 80.6%), bone marrow stimulation (BMS; 47 knees, 75.8%), autologous chondrocyte implantation (ACI; 24 knees, 38.7%) and meniscectomy (29 knees, 46.8%). All were performed during the same operative session as the index MCWDFO. OATS, BMS and ACI frequently overlapped within knees, and only 2 of 62 knees had no concomitant procedure. No individual procedure was significantly associated with signed ΔJLCA (all *p* ≥ 0.29, cluster‐robust standard errors; Table S1).

**Table 1:** Characteristics of the study cohort.

Characteristic	*N* = 62 knees	Missing, *n* (%)
Age, years, mean (SD)	54.24 (11.45)	0 (0)
Sex (male), *n* (%)	20 (32.3)	0 (0)
Side (left), *n* (%)	25 (40.3)	0 (0)
BMI (kg/m^2^), mean (SD)	23.71 (2.96)	0 (0)
Height (m), mean (SD)	1.62 (0.09)	0 (0)
Weight (kg), mean (SD)	62.63 (10.86)	0 (0)
CCI, mean (SD)	0.06 (0.31)	0 (0)
Intraoperative blood loss (mL), mean (SD)	166.77 (166.74)	0 (0)
Operative time (min), mean (SD)	184.37 (54.44)	0 (0)
*Preoperative radiographic characteristics*		
KL classification		
0	0 (0.0)	0 (0)
1	4 (6.5)	0 (0)
2	27 (43.5)	0 (0)
3	24 (38.7)	0 (0)
4	7 (11.3)	0 (0)
Concomitant surgical procedures		
Lateral retinacular release, *n* (%)	42 (67.7)	0 (0)
Meniscectomy, *n* (%)	29 (46.8)	0 (0)
Meniscal repair, *n* (%)	0 (0.0)	0 (0)
Bone marrow stimulation, *n* (%)	47 (75.8)	0 (0)
Osteochondral autograft transplantation, *n* (%)	50 (80.6)	0 (0)
Autologous chondrocyte implantation, *n* (%)	24 (38.7)	0 (0)

*Note*: All concomitant procedures were performed at the time of the index MCWDFO. Procedure categories were not mutually exclusive; percentages were calculated using the 62 included knees as the denominator.

Abbreviations: BMI, body mass index; CCI, Charlson Comorbidity Index; KL, Kellgren–Lawrence classification; MCWDFO, medial closing‐wedge distal femoral osteotomy; SD, standard deviation.

### Radiographic outcomes

Preoperative and 12‐month postoperative radiographic parameters are summarized in Table 2. At 12 months postoperatively, HKAA changed from 4.17 ± 3.49° to −2.05 ± 2.52° (ΔHKAA, −6.21 ± 3.43°), while mLDFA changed from 84.29 ± 2.78° to 89.80 ± 2.59° (ΔmLDFA, 5.51 ± 3.27°). Mean mMPTA changed little (87.22 ± 2.22° to 87.30 ± 2.81°; ΔmMPTA, 0.08 ± 1.63°).

**Table 2:** Changes in radiographic alignment parameters from preoperative assessment to 12‐month follow‐up.

Variable	Preoperative	12‐month postoperative	Change (*Δ*)
JLCA (°), mean (SD)	−1.13 (2.04)	−0.56 (1.61)	0.57 (1.90)
HKAA (°), mean (SD)	4.17 (3.49)	−2.05 (2.52)	−6.21 (3.43)
%MA (%, mean(SD))	65.96 (14.95)	39.13 (11.26)	−26.83 (15.63)
mLDFA (°), mean (SD)	84.29 (2.78)	89.80 (2.59)	5.51 (3.27)
mMPTA (°), mean (SD)	87.22 (2.22)	87.30 (2.81)	0.08 (1.63)
JLO (°), mean (SD)	171.51 (4.15)	177.09 (4.65)	5.59 (4.16)

*Note*: Data are presented as mean (SD). *Δ* was calculated for each knee as the 12‐month postoperative value minus the preoperative value.

Abbreviations: HKAA, hip–knee–ankle angle; JLCA, joint line convergence angle; JLO, joint line obliquity; mLDFA, mechanical lateral distal femoral angle; mMPTA, mechanical medial proximal tibial angle; %MA, percentage of mechanical axis; SD, standard deviation.

Mean signed ΔJLCA was 0.57 ± 1.90° (median, 0.3°; range, −4.6° to 6.9°). Despite the modest mean signed change, the SD of JLCA itself decreased from 2.04° preoperatively to 1.61° at 12 months postoperatively. Markedly negative preoperative JLCA values tended to increase toward 0°, whereas values ≥0° tended to decrease. The postoperative distribution was narrower and more concentrated near 0° (Figure 2).

**Figure 2:**
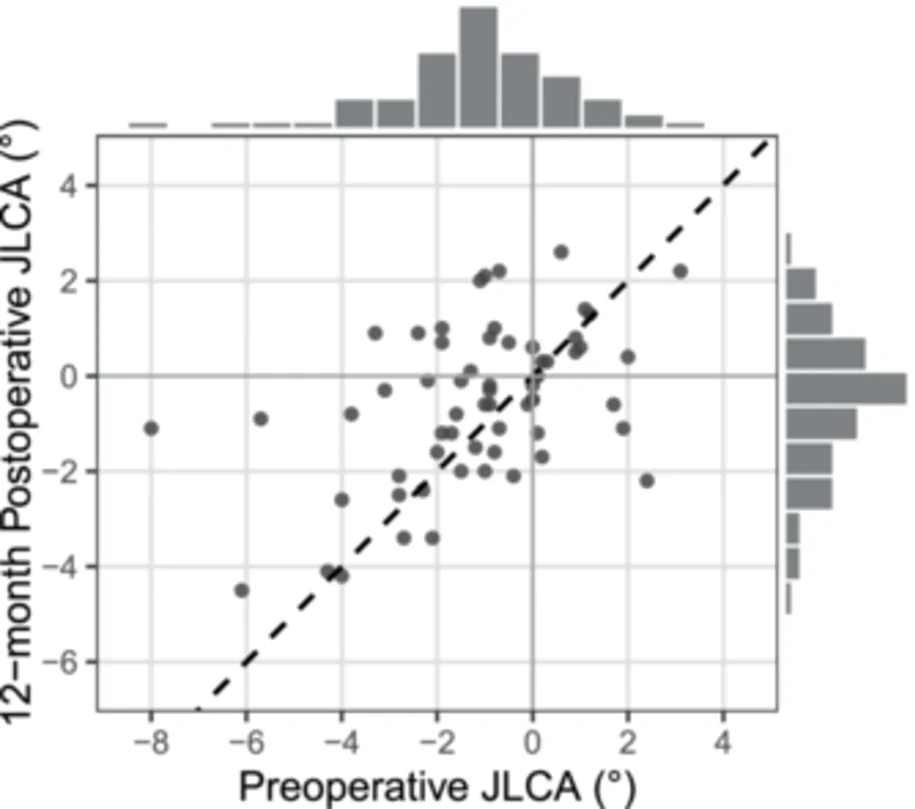
Scatter plot with marginal histograms of preoperative and 12‐month postoperative JLCA. The *x*‐axis shows preoperative JLCA and the *y*‐axis shows 12‐month postoperative JLCA. The upper and right marginal histograms represent the distributions of preoperative and postoperative JLCA, respectively. The dashed diagonal line indicates the line of identity (*y* = *x*). Points above the line indicate increased JLCA after surgery, whereas points below the line indicate decreased JLCA. JLCA, joint line convergence angle.

### Regression analyses of signed ΔJLCA

Univariable and multivariable linear regression results are presented in Table 3. Univariable associations with continuous predictors are illustrated in Figure S1. In univariable analyses, preoperative JLCA was strongly negatively associated with signed ΔJLCA (*B* = −0.62, 95% CI, −0.88 to −0.37; *p* < 0.001). Preoperative mMPTA (*B* = 0.33, 95% CI, 0.06–0.60; *p* = 0.020), preoperative JLO (*B* = 0.19, 95% CI, 0.03–0.34; *p* = 0.024) and age (*B* = 0.04, 95% CI, 0.0002–0.08; *p* = 0.049) were also significantly associated with signed ΔJLCA. Preoperative HKAA (*B* = 0.19, 95% CI, −0.02 to 0.40; *p* = 0.077), preoperative mLDFA (*B* = 0.20, 95% CI, −0.10 to 0.51; *p* = 0.149), BMI (*B* = 0.14, 95% CI, −0.09 to 0.36; *p* = 0.216) and sex (*B* = 0.12, 95% CI, −1.02 to 1.26; *p* = 0.829) were not significantly associated with the outcome.

**Table 3:** Univariable and multivariable associations of preoperative factors with signed ΔJLCA after medial closing‐wedge distal femoral osteotomy (cluster‐robust [CR2] standard errors).

	Univariable regression			Multivariable regression		
Predictor	*B* (crude)	95% CI	*p* value	*B* (adjusted)	95% CI	*p* value
Preoperative JLCA (°)	−0.62	−0.88, −0.37	**<0.001**	−0.56	−0.78, −0.34	**<0.001**
Preoperative HKAA (°)	0.19	−0.02, 0.40	0.077	—	—	—
Preoperative mLDFA (°)	0.20	−0.10, 0.51	0.149	0.06	−0.09, 0.22	0.368
Preoperative mMPTA (°)	0.33	0.06, 0.60	**0.020**	0.23	0.05, 0.41	**0.013**
Preoperative JLO (°)	0.19	0.03, 0.34	**0.024**	—	—	—
Age (years)	0.04	0.00, 0.08	**0.049**	0.02	−0.01, 0.06	0.154
Sex (male)	0.12	−1.02, 1.26	0.829	−0.10	−1.00, 0.80	0.821
BMI (kg/m^2^)	0.14	−0.09, 0.36	0.216	0.11	−0.01, 0.24	0.071

*Note*: 95% CIs and *p* values were calculated using cluster‐robust (CR2) standard errors with Satterthwaite degrees of freedom, clustering on patient (eight patients contributed bilateral knees). The multivariable model yielded an *R* ^2^ of 0.602 and an adjusted *R* ^2^ of 0.559. Female sex was used as the reference category. The lower 95% confidence limit for age in the univariable analysis was 0.0002. Bold values indicate statistical significance (*p* < 0.05).

Abbreviations: *B*, unstandardized regression coefficient; BMI, body mass index; CI, confidence interval; HKAA, hip–knee–ankle angle; JLCA, joint line convergence angle; signed ΔJLCA, 12‐month postoperative JLCA minus preoperative JLCA; JLO, joint line obliquity; mLDFA, mechanical lateral distal femoral angle; mMPTA, mechanical medial proximal tibial angle; —, not included in the multivariable model.

The primary full model included preoperative JLCA, mMPTA, mLDFA, age, sex and BMI based on prior evidence and clinical relevance. HKAA and JLO were excluded because of their mathematical dependence on mLDFA, mMPTA and/or JLCA. Preoperative JLCA (*B* = −0.56, 95% CI, −0.78 to −0.34; *p* < 0.001) and preoperative mMPTA (*B* = 0.23, 95% CI, 0.05–0.41; *p* = 0.013) were independently associated with signed ΔJLCA. Preoperative mLDFA (*B* = 0.06, 95% CI, −0.09 to 0.22; *p* = 0.368), age (*B* = 0.02, 95% CI, −0.01 to 0.06; *p* = 0.154), sex (*B* = −0.10, 95% CI, −1.00 to 0.80; *p* = 0.821) and BMI (*B* = 0.11, 95% CI, −0.01 to 0.24; *p* = 0.071) were not significantly associated with signed ΔJLCA after covariate adjustment and patient‐level clustering. The model yielded *R* ^2 ^= 0.602 and adjusted *R* ^2^ = 0.559.

Supplementary bidirectional AIC‐based stepwise selection excluded mLDFA and sex (Table S2). The reduced model had lower AIC (210.1 vs. 213.1) and similar explanatory performance (*R* ^2^, 0.595 vs. 0.602; adjusted *R* ^2^, 0.567 vs. 0.559) compared with the full model. The full model remained primary, with a maximum VIF of 1.40, indicating no substantial multicollinearity (Table S3).

### Sensitivity analysis

The sensitivity model used the same predictors with 12‐month postoperative JLCA as the outcome (Table S4). Preoperative JLCA was positively associated with 12‐month postoperative JLCA (*B* = 0.44, 95% CI, 0.22–0.66; *p* = 0.001), and preoperative mMPTA (*B* = 0.23, 95% CI, 0.05–0.41; *p* = 0.013) was also independently associated with the outcome, whereas preoperative mLDFA, age, sex and BMI were not significantly associated (all *p* ≥ 0.07). Model *R* ^2^ and adjusted *R* ^2^ were 0.446 and 0.385, respectively. Because postoperative JLCA equals preoperative JLCA plus signed ΔJLCA, the preoperative JLCA coefficient was 1.00 greater than in the primary model (0.44 vs. −0.56), with all other coefficients unchanged.

### Internal validation and predictive performance

Repeated patient‐grouped 10‐fold cross‐validation, with both knees from each patient held out together, yielded an *R* ^2^ of 0.474, an RMSE of 1.36° and an MAE of 1.10°. Cross‐validated *R* ^2^ was 0.128 lower than development‐sample *R* ^2^ (0.474 vs. 0.602). In Figure 3, each knee's *x*‐coordinate represents the mean of 50 out‐of‐fold predictions, one per repeat, generated by models fitted on the other nine folds. Most observations lay near the line of identity, with larger deviations for more extreme signed ΔJLCA values.

**Figure 3:**
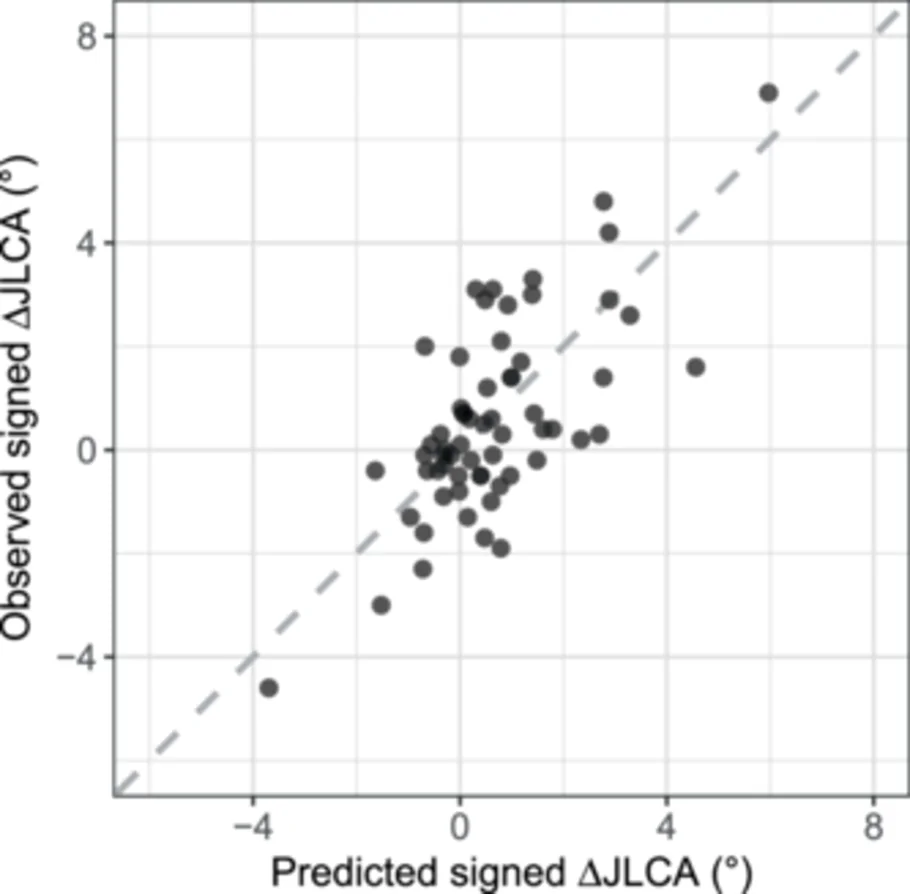
Predicted‐versus‐observed plot of signed ΔJLCA in repeated patient‐grouped 10‐fold cross‐validation. The *x*‐axis shows the predicted signed ΔJLCA (averaged across 50 repeats of patient‐grouped 10‐fold cross‐validation, in which both knees of each bilateral patient were always assigned to the same fold), and the *y*‐axis shows the observed signed ΔJLCA. The dashed line indicates the line of identity (*y* = *x*). For each knee, predictions were generated exclusively from models in which that knee was assigned to the held‐out fold and were averaged across the 50 repeats. JLCA, joint line convergence angle.

Using identical patient‐grouped cross‐validation splits and the same pooling approach, the full model showed better average predictive performance than the JLCA‐only model (*R* ^2^, 0.474 vs. 0.400; RMSE, 1.36° vs. 1.46°; MAE, 1.10° vs. 1.19°; Table S5). The mean differences (full minus JLCA‐only model) were 0.073 for *R* ^2^, −0.092° for RMSE and −0.082° for MAE. The full model yielded higher *R* ^2^ and lower RMSE in 49 of the 50 repeats and lower MAE in all 50 repeats.

## DISCUSSION

The principal findings of this study were a small mean signed ΔJLCA (0.57 ± 1.90°) but substantial between‐knee variability (range, −4.6° to 6.9°) after MCWDFO for valgus knees, and a postoperative JLCA distribution more concentrated around 0°. Preoperative JLCA showed the strongest and most consistent association with signed ΔJLCA. After accounting for within‐patient clustering, preoperative mMPTA was also independently associated in the primary model, whereas preoperative mLDFA, age, sex and BMI were not. Because preoperative JLCA is incorporated into signed ΔJLCA, its inverse association requires cautious interpretation. The model showed moderate internal predictive performance but remains exploratory rather than a precise patient‐specific prediction tool.

Evidence regarding JLCA changes in valgus knees remains limited. Onorato et al. [17] identified femoral correction as a major determinant of absolute ΔJLCA after DFO and reported exploratory thresholds for increased change in valgus knees. Kuwashima et al. [8], however, found no significant mean JLCA change after DLO for valgus deformity. Similarly, mean change was modest in the present cohort, but signed analysis revealed bidirectional variability and a narrower postoperative distribution near 0°, which were obscured by mean or absolute change alone. Maione et al. [14] and Schröter et al. [21] focused on clinical outcomes and correction ranges after MCWDFO rather than the distribution or preoperative correlates of signed JLCA change. The present study extends these findings by characterizing both the direction and magnitude of change.

Preoperative JLCA showed the strongest association in the primary full model and was retained in the AIC‐selected reduced model. Its positive association with 12‐month postoperative JLCA in the sensitivity analysis was directionally consistent with varus‐knee evidence. Previous studies have shown that JLCA change contributes to alignment discrepancy after medial opening‐wedge HTO (MOWHTO) [9], that preoperative soft‐tissue laxity is associated with overcorrection [10], and that JLCA‐adjusted planning may improve correction accuracy [1, 6]. In knees with a preoperative JLCA > 2° undergoing inverted V‐shaped HTO, associations of ΔJLCA with preoperative JLCA and lateral soft‐tissue laxity have also been reported [19]. However, because signed ΔJLCA equals postoperative minus preoperative JLCA, the inverse association may partly reflect mathematical coupling and regression to the mean rather than a biological response. The positive coefficient for preoperative JLCA in the postoperative‐JLCA model shows that the inverse change‐score association is not a negative association with postoperative JLCA. However, because the two model formulations are algebraically related, this sensitivity analysis neither independently validates the primary model nor establishes causality.

A notable finding was the independent association of preoperative mMPTA, representing tibial‐side coronal alignment, but not mLDFA, with signed ΔJLCA after multivariable adjustment. This finding is anatomically plausible because tibial and combined femoral–tibial deformities are common in valgus knees, and tibia valga is associated with higher mMPTA [3, 22]. Maione et al. also reported comparable clinical and mechanical‐axis improvements after MCWDFO for femoral‐ and tibial‐based valgus, despite differences in joint‐line obliquity correction [14]. These observations support separate assessment of femoral and tibial morphology, even when osteotomy is performed at the femur. The mechanism remains uncertain, but load‐dependent interactions among whole‐limb osseous morphology, intra‐articular alignment and soft‐tissue constraint are plausible. The absence of an independent mLDFA association does not imply that femoral correction is unimportant. Its magnitude has been associated with JLCA and JLO changes after DFO [17], and better clinical outcomes have been reported with postoperative mLDFA of 85°–90° than with values > 90° [21]. BMI associations were positive but non‐significant in both univariable and multivariable analyses (multivariable *B* = 0.11, 95% CI, −0.01 to 0.24; *p* = 0.071). Their direction is consistent with a possible load‐dependent effect, as higher BMI and JLCA have been associated with greater intraoperative‐to‐postoperative weight‐bearing alignment discrepancies after MOWHTO [4]; however, this mechanism and its applicability to MCWDFO remain speculative. Mean postoperative HKAA was −2.05°, more varus than the intended target range of 0° to −1°. Intraoperative fine adjustment to avoid residual valgus may have contributed, although this could not be established from the data.

A key strength of this study was its restriction to a single osteotomy type and deformity direction, which reduced procedural heterogeneity. Signed ΔJLCA characterized the direction and magnitude of between‐knee variation, while cluster‐robust standard errors and patient‐grouped cross‐validation accounted for dependence between bilateral knees. The model explained 60.2% of the variance in the development sample, whereas repeated patient‐grouped 10‐fold cross‐validation with predictions pooled across knees within each repeat yielded an *R* ^2^ of 0.474, indicating some optimism in the apparent performance and reinforcing the need for internal validation [23]. The cross‐validated RMSE of 1.36° and MAE of 1.10° indicate errors of approximately 1° and do not support precise patient‐specific prediction. Individual predictions may remain unstable in modest datasets despite acceptable average performance [18]. Because neither study‐specific measurement reliability nor a clinically acceptable tolerance for signed ΔJLCA prediction was established, these errors cannot be considered clinically negligible. The model remains exploratory for identifying preoperative factors and anticipating the direction and approximate magnitude of change.

Clinically, the model may support preoperative assessment by estimating the direction and approximate magnitude of JLCA change and highlighting intra‐articular deformity and tibial‐side alignment. However, predictions should not determine additional correction angles or intraoperative adjustments. The findings apply most directly to primary MCWDFO without additional osteotomy or concomitant ligament repair or reconstruction in settings with similar patient selection, surgical techniques, planning protocols and standardized weight‐bearing full‐length radiographic assessment. Extrapolation to other DFO techniques, HTO, DLO, revision osteotomy, ligament‐unstable knees or post‐traumatic deformity requires caution, particularly because no direct comparison with a varus‐knee cohort was performed.

Several limitations should be acknowledged. First, selection bias, residual confounding and centre‐specific effects cannot be excluded in this retrospective single‐centre study with a relatively small sample. Second, the inclusion of preoperative JLCA in signed ΔJLCA may have inflated their inverse association through mathematical coupling and regression to the mean [26]. The algebraically related postoperative‐JLCA sensitivity model neither independently validates the primary model nor eliminates regression to the mean. Third, the same software and anatomical landmarks were used preoperatively and postoperatively, but the assessors were not blinded to radiographic timing, and formal intraobserver and interobserver reliability and absolute agreement were not assessed. Although mean mMPTA changed little, the SD of ΔmMPTA was 1.63° despite the absence of tibial osteotomy. Differences in radiographic positioning, projection and landmark identification may have contributed; this variability should not be assumed to represent true changes in tibial osseous alignment [2, 5, 11]. Because mMPTA and JLCA measurement reliability may vary according to anatomical and radiographic conditions [11], the contribution of measurement error to observed variability and prediction error remains uncertain, and small individual changes cannot be assumed to exceed measurement error. The findings should therefore remain descriptive and exploratory, not a basis for individual correction adjustments. Fourth, despite a uniform femoral osteotomy without additional osteotomy or ligament repair or reconstruction, deformity etiologies and concomitant procedures varied. Exploratory procedure‐specific analyses could not isolate independent effects because procedures overlapped; specific combinations and treated compartments were not evaluated separately. Fifth, cluster‐robust standard errors and patient‐grouped cross‐validation did not address unmeasured patient‐level factors shared by bilateral knees. Finally, the model underwent repeated patient‐grouped 10‐fold cross‐validation but lacks external validation. Independent multicenter validation is required before clinical implementation [15].

## CONCLUSION

Signed ΔJLCA after MCWDFO for valgus knees showed substantial between‐knee variability despite a small mean change, with postoperative JLCA clustering more closely around 0°. Preoperative JLCA showed the strongest and most consistent association with signed ΔJLCA, although mathematical coupling warrants caution. Preoperative mMPTA was also independently associated with signed ΔJLCA, whereas preoperative mLDFA, age, sex and BMI were not significantly associated. The multivariable model showed moderate internal predictive performance but remains exploratory and should not be used for precise patient‐specific prediction or to determine additional correction angles. Preoperative assessment may benefit from considering intra‐articular deformity and tibial‐side alignment alongside femoral correction. External validation and formal radiographic reliability assessment are required before clinical implementation.

## AUTHOR CONTRIBUTIONS

**Yuki Kato**: Conceptualization; methodology; investigation; supervision; writing—original draft; writing—review and editing. **Ken Ichikawa**: Data curation; formal analysis; investigation. **Hiroki Matsui**: Formal analysis; methodology; writing—review and editing. **Shuzo Takazawa**: Investigation. **Shunsuke Koga**: Investigation. **Shin Yamada**: Investigation. **Asami Kiuchi**: Investigation. **Akira Ryu**: Investigation. **Hiroshi Ohuchi**: Supervision. The statistical reanalysis for the revised manuscript was performed by Ken Ichikawa.

## FUNDING INFORMATION

This work was supported by Osferion Biomaterials Corp., Japan, under a collaborative research agreement with Kameda Medical Center. The article processing charge was also covered by this funding. The funder had no role in the study design; data collection, analysis, or interpretation; manuscript preparation; or the decision to submit the manuscript for publication.

## CONFLICT OF INTEREST STATEMENT

This study received research funding from Osferion Biomaterials Corp., Japan, paid to Kameda Medical Center under a collaborative research agreement. The authors declare no other conflicts of interest.

## ETHICS STATEMENT

This study was conducted in accordance with the Declaration of Helsinki and was approved by the Institutional Review Board of Kameda Medical Center (approval no. 24‐074). The requirement for individual written informed consent was waived because of the retrospective study design. Information regarding the study was disclosed through an institutional opt‐out procedure, and patients were given the opportunity to decline the use of their data for research.

## Supporting information


**Supplementary Figure 1. Univariable associations between signed ΔJLCA and continuous candidate predictor variables.** Each point represents one knee. Solid lines indicate fitted univariable linear regression lines, and shaded bands indicate the corresponding 95% confidence intervals. The horizontal dashed line represents no change in JLCA (signed ΔJLCA = 0°). Signed ΔJLCA was calculated as the 12‐month postoperative JLCA minus the preoperative JLCA. BMI, body mass index; HKAA, hip–knee–ankle angle; JLCA, joint line convergence angle; JLO, joint line obliquity; mLDFA, mechanical lateral distal femoral angle; mMPTA, mechanical medial proximal tibial angle.

Supporting file 1.

Supporting file 2.

Supporting file 3.

Supporting file 4.

Supporting file 5.

Supporting file 6.

## Data Availability

The data supporting the findings of this study are stored securely within the authors' institution and are not publicly available because of institutional data protection policies.
