# Supplementary material for: Bidirectional joint line convergence angle changes and their preoperative predictors 12 months after medial closing‐wedge distal femoral osteotomy for valgus knees: A single‐centre exploratory study
Source: J Exp Orthop. 2026 Nov 3;13(4):e70975. doi: 10.1002/jeo2.70975 (PMC13730025; doi:10.1002/jeo2.70975)
Supplement: Supplementary file 1 [file JEO2-13-e70975-s001.docx]

**Supplementary Table 1. Association between concomitant procedures and signed ΔJLCA**

| **Procedure** | **Knees, n (%)** | **Mean difference (°)** | **95% CI** | **p value** |
| --- | --- | --- | --- | --- |
| Lateral retinacular release | 42 (67.7) | −0.18 | −1.44, 1.08 | 0.772 |
| Osteochondral autograft transplantation | 50 (80.6) | −0.39 | −2.25, 1.48 | 0.664 |
| Bone marrow stimulation | 47 (75.8) | −0.12 | −1.65, 1.42 | 0.877 |
| Autologous chondrocyte implantation | 24 (38.7) | 0.53 | −0.47, 1.54 | 0.290 |
| Meniscectomy | 29 (46.8) | 0.12 | −0.86, 1.10 | 0.802 |

CI, confidence interval. Mean difference indicates the difference in signed ΔJLCA between knees that did and did not undergo the indicated procedure (performed minus not performed), estimated by linear regression. 95% CIs and p values were calculated using cluster-robust (CR2) standard errors with Satterthwaite degrees of freedom, clustering on patient (8 patients contributed bilateral knees). Because concomitant procedures were frequently combined (only 2 of 62 knees underwent isolated MCWDFO without any concomitant procedure), these comparisons should be interpreted as exploratory and do not isolate the independent effect of any single procedure.
