# Supplementary material for: Bidirectional joint line convergence angle changes and their preoperative predictors 12 months after medial closing‐wedge distal femoral osteotomy for valgus knees: A single‐centre exploratory study
Source: J Exp Orthop. 2026 Nov 3;13(4):e70975. doi: 10.1002/jeo2.70975 (PMC13730025; doi:10.1002/jeo2.70975)
Supplement: Supplementary file 2 [file JEO2-13-e70975-s002.docx]

STROBE Statement — Cohort studies

**Bidirectional Joint Line Convergence Angle Changes and Their Preoperative Predictors 12 Months After Medial Closing-Wedge Distal Femoral Osteotomy for Valgus Knees: A Single-Center Exploratory Study**

Reference version: “Revised manuscript 2026-10-9.pdf” (23 pages). Locations use the page and line numbers printed in that manuscript, not the pagination of the system-generated submission package. Table and figure bodies without line numbers are identified by number and page. Supplementary files and the non-anonymized title page are identified separately.

# Title, abstract, introduction, and study population

| **Item / topic** | **STROBE recommendation** | **Page / printed lines** | **Reporting details / applicability** |
| --- | --- | --- | --- |
| **1(a) Title and abstract** | Indicate the study’s design with a commonly used term in the title or the abstract. | p. 1, lines 13–15 | The Abstract identifies a retrospective single-center cohort. The title identifies the exploratory nature of the study. |
| **1(b) Title and abstract** | Provide in the abstract an informative and balanced summary of what was done and what was found. | p. 1, lines 8–34; p. 2, lines 35–38 | Structured summary of the purpose, cohort, measurements, analyses, numerical findings, mathematical coupling, exploratory prediction, and need for further validation. |
| **2 Background / rationale** | Explain the scientific background and rationale for the investigation being reported. | p. 3, lines 46–76 | Describes prior osteotomy and JLCA literature and the evidence gap concerning signed JLCA changes after MCWDFO for valgus knees. |
| **3 Objectives** | State specific objectives, including any prespecified hypotheses. | p. 3, lines 78–79; p. 4, lines 80–85 | States the hypotheses, primary descriptive objective, and secondary exploratory prediction objective. |
| **4 Study design** | Present key elements of study design early in the paper. | p. 4, lines 91–94 | Single-center retrospective observational cohort of consecutive operated knees, with preoperative and 12-month assessment. |
| **5 Setting** | Describe the setting, locations, and relevant dates, including periods of recruitment, exposure, follow-up, and data collection. | p. 4, lines 104–108, 111; p. 5, lines 112–123; p. 6, lines 162–168 | Reports the Japanese referral-center setting, October 2017–December 2024 surgical period, data sources, and routine 12-month follow-up. A separate calendar period for retrospective data extraction is not stated. |
| **6(a) Participants** | Give the eligibility criteria, and the sources and methods of selection of participants. Describe methods of follow-up. | p. 4, line 111; p. 5, lines 112–123; p. 6, lines 162–180 | Consecutive MCWDFO knees were identified through institutional clinical records. Inclusion/exclusion criteria, permitted concomitant procedures, and routine 12-month weight-bearing radiographic assessment are described. |
| **6(b) Participants** | For matched studies, give matching criteria and number of exposed and unexposed. | N/A; design described at p. 4, lines 91–94 | No matched study design or matched exposed/unexposed groups are reported. |

# Methods — Variables, measurement, and statistical methods

| **Item / topic** | **STROBE recommendation** | **Page / printed lines** | **Reporting details / applicability** |
| --- | --- | --- | --- |
| **7 Variables** | Clearly define all outcomes, exposures, predictors, potential confounders, and effect modifiers. Give diagnostic criteria, if applicable. | p. 5, lines 115–116, 139–146; p. 6, lines 147–159; p. 7, lines 207–216; p. 8, lines 217–218 | Defines signed ΔJLCA, the JLCA and HKAA sign conventions, JLO, candidate predictors, and adjustment variables. Valgus eligibility is defined by HKAA ≥0°. No effect-modifier analyses are described. |
| **8* Data sources / measurement** | For each variable of interest, give sources of data and details of methods of assessment (measurement). Describe comparability of assessment methods if there is more than one group. | p. 6, lines 162–182; p. 7, lines 183–184 | Identifies the clinical database, records, imaging protocol, software, and two experienced assessors. The same definitions and landmarks were used at both time points. Non-blinding and absence of formal intra-/interobserver reliability and absolute-agreement assessment are explicitly stated. |
| **9 Bias** | Describe any efforts to address potential sources of bias. | p. 7, lines 187–195; p. 8, lines 229–247; p. 14, lines 427–449 | Consecutive inclusion, routinely collected data, a predefined measurement protocol, patient-clustered inference, and patient-grouped validation are described. Residual selection, measurement, and other biases are acknowledged. |
| **10 Study size** | Explain how the study size was arrived at. | p. 7, lines 198–204; p. 9, lines 254–261 | All eligible knees with available data in the defined period were included; no a priori sample-size calculation was performed. Final sample: 62 knees from 54 patients. |
| **11 Quantitative variables** | Explain how quantitative variables were handled in the analyses. If applicable, describe which groupings were chosen and why. | p. 7, lines 207–216; p. 8, lines 217–223 | Continuous variables were retained as continuous measures. Angle signs, change-score calculations, and descriptive summaries are specified. |
| **12(a) Statistical methods** | Describe all statistical methods, including those used to control for confounding. | p. 8, lines 221–248; p. 6, lines 147–159 | Describes univariable/multivariable regression, six-variable primary model, covariate rationale, collinearity checks, CR2 inference with Satterthwaite degrees of freedom, sensitivity analyses, and repeated patient-grouped validation. |
| **12(b) Statistical methods** | Describe any methods used to examine subgroups and interactions. | p. 8, lines 236–238; p. 14, lines 441–445 | Procedure-specific comparisons used separate linear regressions. No interaction analyses are described. Specific procedure combinations and treated compartments were not evaluated separately. |

# Methods and results — Data completeness and participants

| **Item / topic** | **STROBE recommendation** | **Page / printed lines** | **Reporting details / applicability** |
| --- | --- | --- | --- |
| **12(c) Statistical methods** | Explain how missing data were addressed. | p. 5, lines 120–123; p. 9, lines 259–261; Table 1, p. 18 | Availability of required clinical and radiographic data was an eligibility requirement. All 62 analyzed knees had complete study data. Table 1 reports zero missing values for the listed characteristics; no imputation procedure is described. |
| **12(d) Statistical methods** | If applicable, explain how loss to follow-up was addressed. | p. 5, lines 120–123; p. 9, lines 254–261; Figure 1, p. 21, legend lines 583–585 | The analyzed knees all had the required 12-month data. No losses to follow-up are reported among included knees, and no attrition-adjustment method is described. |
| **12(e) Statistical methods** | Describe any sensitivity analyses. | p. 8, lines 234–247 | Specifies the postoperative-JLCA sensitivity model, supplementary bidirectional AIC selection, individual-procedure regressions, and full-versus-JLCA-only comparison using identical validation splits. |
| **13(a)* Participants** | Report numbers of individuals at each stage of study—eg numbers potentially eligible, examined for eligibility, confirmed eligible, included in the study, completing follow-up, and analysed. | p. 9, lines 254–261; Figure 1, p. 21, legend lines 583–585 | Reports knee-level flow (68 screened; 1 excluded; 67 remaining; 5 further exclusions; 62 analyzed), complete 12-month data, and 54 unique patients including 8 bilateral cases (16 knees). |
| **13(b)* Participants** | Give reasons for non-participation at each stage. | p. 9, lines 255–261; Figure 1, p. 21 | Exclusions: concomitant DLO (1), previous intra-articular fracture surgery (4), and prior knee osteotomy (1). No exclusions for prior LCL surgery or missing study variables are reported. |
| **13(c)* Participants** | Consider use of a flow diagram. | Figure 1, p. 21, legend lines 583–585; p. 9, lines 254–261 | A flow diagram depicts screening, reasons for exclusion, and the analyzed population. |
| **14(a)* Descriptive data** | Give characteristics of study participants (eg demographic, clinical, social) and information on exposures and potential confounders. | p. 9, lines 263–274; Table 1, p. 18; Table 2, p. 19 | Reports demographics, KL grade, operative characteristics, concomitant procedures, and preoperative radiographic parameters. Procedure counts are knee-level and categories overlap. |
| **14(b)* Descriptive data** | Indicate number of participants with missing data for each variable of interest. | p. 9, lines 259–261; Table 1, p. 18 (Missing, n [%] column) | All study variables are reported as complete for the 62 analyzed knees. Table 1 provides variable-level zero-missing counts for its listed characteristics. |
| **14(c)* Descriptive data** | Summarise follow-up time (eg, average and total amount). | p. 6, lines 166–168; p. 9, lines 259–261, 277–284 | Partly reported: the routine 12-month assessment and complete data at that assessment are specified. The actual follow-up-time distribution and total follow-up time are not reported; these are not inferred from the scheduled visit. |

# Results — Outcomes and analyses

| **Item / topic** | **STROBE recommendation** | **Page / printed lines** | **Reporting details / applicability** |
| --- | --- | --- | --- |
| **15* Outcome data** | Report numbers of outcome events or summary measures over time. | p. 9, lines 277–284; p. 10, lines 285–286; Table 2, p. 19; Figure 2, p. 22, legend lines 589–595 | Reports preoperative and 12-month angle summaries and paired changes. Signed ΔJLCA: mean 0.57 ± 1.90°, median 0.3°, range −4.6° to 6.9°. The outcome is continuous, not an event count. |
| **16(a) Main results** | Give unadjusted estimates and, if applicable, confounder-adjusted estimates and their precision (eg, 95% confidence interval). Make clear which confounders were adjusted for and why they were included. | p. 10, lines 289–306; Table 3, p. 20; p. 6, lines 147–159; p. 8, lines 224–233 | Reports unadjusted and adjusted coefficients, 95% CIs, and p values. The primary covariates and their rationale are specified. Patient-clustered CR2 inference is distinguished from the ordinary least squares point estimates. |
| **16(b) Main results** | Report category boundaries when continuous variables were categorized. | N/A; p. 7, lines 207–216; p. 8, lines 217–218 | Continuous predictors were not categorized. The HKAA eligibility threshold and descriptive KL grades are not data-driven predictor categories. |
| **16(c) Main results** | If relevant, consider translating estimates of relative risk into absolute risk for a meaningful time period. | N/A; outcome definition: p. 5, lines 139–143 | The outcome is angular change analyzed by linear regression; no relative-risk estimates are reported. |
| **17 Other analyses** | Report other analyses done—eg analyses of subgroups and interactions, and sensitivity analyses. | p. 9, lines 268–274; p. 10, lines 307–319; p. 11, lines 320–336; Supplementary Tables 1–5, separate files, each p. 1 | Reports procedure comparisons, AIC selection, VIFs, the postoperative-JLCA model, and internal predictive performance including the JLCA-only comparison. Supplementary Tables 1–5 are referenced as separate files, not pages of the main manuscript. |

**Supplementary-file locations (local pagination)**

Supplementary Table 1: separate file, p. 1 — concomitant-procedure comparisons and explanatory footnote.

Supplementary Table 2: separate file, p. 1 — primary full and AIC-selected models and explanatory footnote.

Supplementary Table 3: separate file, p. 1 — variance inflation factors and explanatory footnote.

Supplementary Table 4: separate file, p. 1 — postoperative-JLCA sensitivity model and explanatory footnote.

Supplementary Table 5: separate file, p. 1 — cross-validated full-versus-JLCA-only performance and explanatory footnote. Table bodies have no printed line numbers.

# Discussion and other information

| **Item / topic** | **STROBE recommendation** | **Page / printed lines** | **Reporting details / applicability** |
| --- | --- | --- | --- |
| **18 Key results** | Summarise key results with reference to study objectives. | p. 11, lines 341–349; p. 14, lines 453–461; p. 15, lines 462–463 | Summarizes bidirectional change, a small mean with substantial between-knee variation, associations with preoperative JLCA and mMPTA, and exploratory predictive performance. |
| **19 Limitations** | Discuss limitations of the study, taking into account sources of potential bias or imprecision. Discuss both direction and magnitude of any potential bias. | p. 13, lines 425–426; p. 14, lines 427–449; p. 13, lines 400–413 | Addresses selection and residual confounding, mathematical coupling/regression to the mean, non-blinded and untested measurement reliability, overlapping procedures, shared patient-level factors, and absent external validation. Possible inflation of the inverse association is described; measurement-bias magnitude remains unquantified. |
| **20 Interpretation** | Give a cautious overall interpretation of results considering objectives, limitations, multiplicity of analyses, results from similar studies, and other relevant evidence. | p. 11, lines 351–354; p. 12, lines 355–390; p. 13, lines 391–423; p. 14, lines 453–461; p. 15, lines 462–463 | Compares prior studies, discusses alternative explanations and algebraic coupling, and limits claims to descriptive findings and exploratory associations. Clinical use is not established. The primary/supplementary distinction is also specified in Results (p. 10, lines 307–311). |
| **21 Generalisability** | Discuss the generalisability (external validity) of the study results. | p. 13, lines 415–423; p. 14, lines 447–449 | Limits applicability to similar primary MCWDFO cohorts and clinical/imaging protocols. Extrapolation to other procedures or case mixes is cautioned, and external multicenter validation is requested. |
| **22 Funding** | Give the source of funding and the role of the funders for the present study and, if applicable, for the original study on which the present article is based. | Separate non-anonymized Title Page, p. 2: Funding statement; Conflict of interest disclosure (no printed line numbers) | The funder, institutional research support, article-processing-charge support, and absence of funder involvement in study conduct, analysis, writing, or submission are disclosed in the separate title page. No funding statement appears in the anonymized main manuscript. |

N/A means not applicable to the design or analyses reported. A reporting limitation is not reclassified as N/A; for example, the actual follow-up-time distribution is not supplied. No unperformed reliability assessment, subgroup analysis, or external validation is implied by this checklist.

* The STROBE cohort checklist requests separate reporting for exposed and unexposed groups where relevant. The primary analysis here concerns a single surgical cohort and continuous preoperative predictors. Exploratory procedure-specific comparisons are reported separately.

Source convention for item 22: the funding location was verified in the separately submitted title page reproduced in jeo.202600911_R1.pdf, PDF p. 29 (local Title Page p. 2). Its pagination is distinct from the 23-page manuscript used for items 1–21.

STROBE source: von Elm E, Altman DG, Egger M, et al. The Strengthening the Reporting of Observational Studies in Epidemiology (STROBE) Statement: guidelines for reporting observational studies. PLoS Med. 2007;4(10):e296. doi:10.1371/journal.pmed.0040296. Cohort checklist: STROBE Initiative. The original checklist recommendations and numbering are retained; manuscript locations and reporting notes have been added.
