# Supplementary material for: Bidirectional joint line convergence angle changes and their preoperative predictors 12 months after medial closing‐wedge distal femoral osteotomy for valgus knees: A single‐centre exploratory study
Source: J Exp Orthop. 2026 Nov 3;13(4):e70975. doi: 10.1002/jeo2.70975 (PMC13730025; doi:10.1002/jeo2.70975)
Supplement: Supplementary file 3 [file JEO2-13-e70975-s003.docx]

**Supplementary Table 5. Cross-validated predictive performance of the full model and the preoperative JLCA-only model**

| **Metric** | **Full model** | **JLCA-only model** | **Mean difference** | **No. of repeats favoring full model** |
| --- | --- | --- | --- | --- |
| R² | 0.474 | 0.400 | 0.073 | 49/50 |
| RMSE (°) | 1.36 | 1.46 | −0.092 | 49/50 |
| MAE (°) | 1.10 | 1.19 | −0.082 | 50/50 |
| Both models were evaluated using identical repeated patient-grouped 10-fold cross-validation splits (50 repeats). Within each repeat, performance metrics were calculated from pooled out-of-fold predictions for all 62 knees and then averaged across repeats. The full model included preoperative JLCA, mMPTA, mLDFA, age, sex, and BMI. Mean difference is the full model minus the JLCA-only model. JLCA, joint line convergence angle; mMPTA, mechanical medial proximal tibial angle; mLDFA, mechanical lateral distal femoral angle; BMI, body mass index; RMSE, root mean squared error; MAE, mean absolute error. | | | | |
