# Supplementary material for: Bidirectional joint line convergence angle changes and their preoperative predictors 12 months after medial closing‐wedge distal femoral osteotomy for valgus knees: A single‐centre exploratory study
Source: J Exp Orthop. 2026 Nov 3;13(4):e70975. doi: 10.1002/jeo2.70975 (PMC13730025; doi:10.1002/jeo2.70975)
Supplement: Supplementary file 4 [file JEO2-13-e70975-s004.docx]

**Supplementary Table 4. Sensitivity analysis using 12-month postoperative JLCA as the dependent variable**

| **Predictor** | **B** | **95% CI** | **p value** |
| --- | --- | --- | --- |
| Preoperative JLCA (°) | 0.44 | 0.22, 0.66 | **0.001** |
| Preoperative mMPTA (°) | 0.23 | 0.05, 0.41 | **0.013** |
| Preoperative mLDFA (°) | 0.06 | −0.09, 0.22 | 0.368 |
| Age (years) | 0.02 | −0.01, 0.06 | 0.154 |
| Sex (male) | −0.10 | −1.00, 0.80 | 0.821 |
| BMI (kg/m²) | 0.11 | −0.01, 0.24 | 0.071 |

B, unstandardized regression coefficient; CI, confidence interval; JLCA, joint line convergence angle; mMPTA, mechanical medial proximal tibial angle; mLDFA, mechanical lateral distal femoral angle; BMI, body mass index. The same predictors as in the primary model were included, with 12-month postoperative JLCA, rather than signed ΔJLCA, as the dependent variable. Because 12-month postoperative JLCA equals preoperative JLCA plus signed ΔJLCA, the coefficient for preoperative JLCA was expected to be 1.00 greater than that in the primary model, whereas the coefficients for the remaining predictors were unchanged. Female sex was used as the reference category. The model yielded an R² of 0.446 and an adjusted R² of 0.385.
