# Supplementary material for: Bidirectional joint line convergence angle changes and their preoperative predictors 12 months after medial closing‐wedge distal femoral osteotomy for valgus knees: A single‐centre exploratory study
Source: J Exp Orthop. 2026 Nov 3;13(4):e70975. doi: 10.1002/jeo2.70975 (PMC13730025; doi:10.1002/jeo2.70975)
Supplement: Supplementary file 5 [file JEO2-13-e70975-s005.docx]

**Supplementary Figure 1. Univariable associations between signed ΔJLCA and continuous candidate predictor variables**

Each point represents one knee. Solid lines indicate fitted univariable linear regression lines, and shaded bands indicate the corresponding 95% confidence intervals. The horizontal dashed line represents no change in JLCA (signed ΔJLCA = 0°). Signed ΔJLCA was calculated as the 12-month postoperative JLCA minus the preoperative JLCA. BMI, body mass index; HKAA, hip–knee–ankle angle; JLCA, joint line convergence angle; JLO, joint line obliquity; mLDFA, mechanical lateral distal femoral angle; mMPTA, mechanical medial proximal tibial angle.
