# Supplementary material for: Bidirectional joint line convergence angle changes and their preoperative predictors 12 months after medial closing‐wedge distal femoral osteotomy for valgus knees: A single‐centre exploratory study
Source: J Exp Orthop. 2026 Nov 3;13(4):e70975. doi: 10.1002/jeo2.70975 (PMC13730025; doi:10.1002/jeo2.70975)
Supplement: Supplementary file 6 [file JEO2-13-e70975-s006.docx]

**Supplementary Table 2. Comparison of full model and AIC-selected model.**

| Model | Variables included | AIC | R² | Adjusted R² |
| --- | --- | --- | --- | --- |
| Primary full model | Preoperative JLCA, preoperative mMPTA, preoperative mLDFA, age, sex, BMI | 213.1 | 0.602 | 0.559 |
| AIC-selected reduced model | Preoperative JLCA, preoperative mMPTA, age, BMI | 210.1 | 0.595 | 0.567 |

The primary full model was specified on the basis of prior evidence and clinical relevance and was used for primary inference. The AIC-selected reduced model was derived using bidirectional stepwise variable selection and was examined as a supplementary analysis. A lower AIC indicates better relative model fit after accounting for model complexity.

AIC, Akaike information criterion; JLCA, joint line convergence angle; mMPTA, mechanical medial proximal tibial angle; mLDFA, mechanical lateral distal femoral angle; BMI, body mass index.
