# Supplementary material for: Bidirectional joint line convergence angle changes and their preoperative predictors 12 months after medial closing‐wedge distal femoral osteotomy for valgus knees: A single‐centre exploratory study
Source: J Exp Orthop. 2026 Nov 3;13(4):e70975. doi: 10.1002/jeo2.70975 (PMC13730025; doi:10.1002/jeo2.70975)
Supplement: Supplementary file 7 [file JEO2-13-e70975-s007.docx]

**Supplementary Table 3. Variance inflation factors for variables included in the primary multivariable model**

| Variable | VIF |
| --- | --- |
| Preoperative JLCA (°) | 1.06 |
| Preoperative mMPTA (°) | 1.18 |
| Preoperative mLDFA (°) | 1.27 |
| Age (years) | 1.32 |
| Sex | 1.40 |
| BMI (kg/m²) | 1.06 |

JLCA, joint line convergence angle; mMPTA, mechanical medial proximal tibial angle; mLDFA, mechanical lateral distal femoral angle; BMI, body mass index; VIF, variance inflation factor. All VIFs were <5, with a maximum value of 1.40, indicating no substantial multicollinearity.
